# Glycyrrhizin-Induced Pseudoaldosteronism Contributing to Decompensated Heart Failure With Preserved Ejection Fraction

**DOI:** 10.7759/cureus.116676

**Published:** 2026-09-21

**Authors:** Satoshi Kurisu, Hitoshi Fujiwara

**Affiliations:** 1 Department of Cardiology, National Hospital Organization (NHO) Hiroshimanishi Medical Center, Otake, JPN

**Keywords:** adverse effects, elderly patients, herbal drugs, persistent hypokalemia, renin-angiotensin-aldosterone system

## Abstract

Glycyrrhizin, a component of licorice-containing medications such as Shakuyakukanzoto, can cause pseudoaldosteronism, characterized by hypokalemia and hypertension. However, its potential role in contributing to decompensated heart failure with preserved ejection fraction (HFpEF) has rarely been described.

We report an elderly man who presented with dyspnea, peripheral edema, and pleural effusion after more than 10 years of Shakuyakukanzoto use. Electrocardiography showed atrial fibrillation, and echocardiography showed preserved left ventricular ejection fraction, left atrial enlargement, and echocardiographic findings suggestive of elevated left ventricular filling pressure, supporting the diagnosis of HFpEF. At presentation, we suspected pseudoaldosteronism based on the combination of hypokalemia, inappropriate renal potassium loss, and long-term use of Shakuyakukanzoto. Plasma renin activity and plasma aldosterone concentration were suppressed, supporting the diagnosis. Discontinuation of Shakuyakukanzoto, together with potassium supplementation, diuretic therapy, and spironolactone, resulted in resolution of hypokalemia and congestive findings and allowed reduction of antihypertensive medications. In conclusion, glycyrrhizin-induced pseudoaldosteronism may contribute to decompensated HFpEF through sodium and water retention in patients with limited cardiac tolerance to volume loading.

## Introduction

Herbal medicines are widely used in Japan for a variety of clinical conditions, and many of these medicines contain licorice [1-5]. Glycyrrhizin in licorice inhibits 11β-hydroxysteroid dehydrogenase type 2 (11β-HSD2), allowing cortisol to activate mineralocorticoid receptors and resulting in sodium retention and potassium excretion, potentially leading to volume expansion [1-5]. This pathophysiological process is referred to as pseudoaldosteronism, which clinically resembles primary aldosteronism but is characterized by suppressed plasma renin activity (PRA) and plasma aldosterone concentration (PAC).

Pseudoaldosteronism is a well-recognized adverse effect of glycyrrhizin-containing medications, including Shakuyakukanzoto [4]. The resulting sodium and water retention may be particularly relevant in patients with a predisposition to heart failure with preserved ejection fraction (HFpEF), in whom even relatively small increases in volume may lead to disproportionate elevations in cardiac filling pressures and promote pulmonary and systemic congestion despite preserved systolic function [6]. However, HFpEF as a clinical manifestation of pseudoaldosteronism has rarely been reported.

In this report, we describe an elderly man in whom glycyrrhizin-induced pseudoaldosteronism contributed to decompensated HFpEF.

## Case presentation

A 94-year-old man with hypertension and atrial fibrillation, but no history of heart failure, presented to the emergency department with progressive dyspnea for one week. His medications included azilsartan 20 mg/day, amlodipine 5 mg/day, bisoprolol 2.5 mg/day, and edoxaban 30 mg/day. He had also been taking Shakuyakukanzoto 5 g/day for muscle cramps for more than 10 years. On admission, his blood pressure was 160/88 mmHg, heart rate was 96 beats/min, and respiratory rate was 28 breaths/min. His oxygen saturation was 98% while receiving oxygen at 4 L/min. Physical examination revealed jugular venous distension and bilateral lower-extremity edema. Laboratory investigations revealed hypokalemia (serum potassium, 3.2 mmol/L) and an elevated N-terminal pro-B-type natriuretic peptide (NT-proBNP) level of 2,828 pg/mL. Serum creatinine was 0.97 mg/dL, with an estimated glomerular filtration rate of 54.4 mL/min/1.73 m², calculated using the Japanese equation. Spot urinary potassium concentration measured concurrently with blood sampling was 27 mmol/L before the initiation of diuretic therapy, despite hypokalemia, suggesting inappropriate renal potassium loss (Table 1).

**Table 1:** Laboratory data eGFR, estimated glomerular filtration rate; NT-proBNP, N-terminal pro-B-type natriuretic peptide; PAC, plasma aldosterone concentration; PRA, plasma renin activity.

Variable	Day 1	Day 9	Day 13	Day 22	Day 28	Reference range
Blood count						
White blood cell count (× 10^3^/µL)	9.2	6.6	3.8	4.7	-	3.3-8.6
Red blood cell count (× 10^6^/µL)	3.7	3.54	3.43	3.68	-	3.86-4.92
Hemoglobin (g/dL)	12.2	11.9	11.3	12.1	-	11.6-14.8
Platelet count (× 10^3^/µL)	196	257	254	227	-	158-348
Blood chemistry						
Total bilirubin (mg/dL)	0.8	0.7	-	-	0.7	0.4-1.5
Aspartate aminotransferase (U/L)	27	27	-	-	23	13-30
Alanine aminotransferase (U/L)	13	16	-	-	16	7-23
Lactate dehydrogenase (U/L)	205	-	-	-	157	124-222
Alkaline phosphatase (U/L)	98	96	-	-	-	38-113
γ-glutamyl transferase (U/L)	123	119	-	-	71	9-32
Total protein (g/dL)	7.3	6.7	-	-	-	6.6-8.1
Albumin (g/dL)	3.7	3.2	-	-	-	4.1-5.1
Blood urea nitrogen (mg/dL)	12	10	12	11	14	8-20
Creatinine (mg/dL)	0.97	0.89	1.01	0.93	0.92	0.46-0.79
eGFR (mL/min/1.73 m^2^)	54.4	59.8	52.1	57	57.7	
Sodium (mmol/L)	127	133	130	132	133	138-145
Potassium (mmol/L)	3.2	3.9	3.7	4.3	4.3	3.6-4.8
Chloride (mmol/L)	89	86	88	92	95	101-108
Calcium (mg/dL)	8.4	-	-	-	-	8.8-10.1
Magnesium (mg/dL)	1.4	-	-	-	-	1.8-2.6
C-reactive protein (mg/dL)	5	-	3.2	0.99	-	<0.14
Glucose (mg/dL)	164	147	129	-	-	
NT-proBNP (pg/mL)	2,828	-	-	1,889	-	<126
Troponin I (pg/mL)	46.2	-	-	-	-	0-26.2
PAC (pg/mL)	<4.0	-	-	-	-	4.0-82.1
PRA (ng/mL/h)	0.4	-	-	-	-	0.2-2.3 (supine)
Spot urinalysis						
Sodium (mmol/L)	30	-	20	-	-	
Potassium (mmol/L)	27	-	11	-	-	
Chloride (mmol/L)	38	-	21	-	-	

Chest radiography revealed cardiomegaly, left pleural calcification, and bilateral pulmonary congestion (Figure 1A).

**Figure 1:**
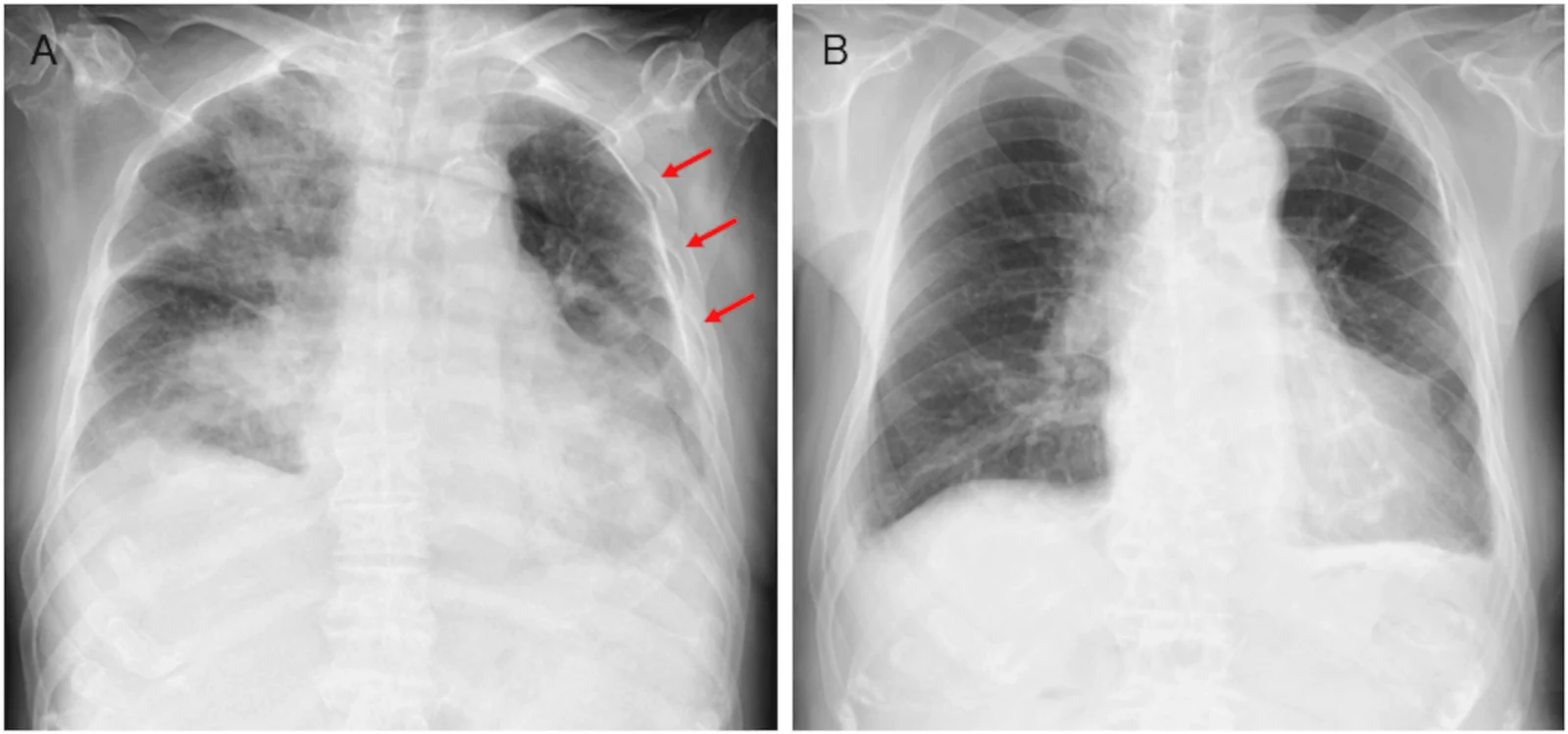
Chest radiographs Chest radiography revealed cardiomegaly, left pleural calcification (arrows), and bilateral pulmonary congestion (A). Three weeks later, pleural effusion and pulmonary congestion resolved on chest radiography (B).

Subsequent chest computed tomography revealed bilateral pleural effusions, greater on the right, accompanied by pulmonary congestion (Figure 2).

**Figure 2:**
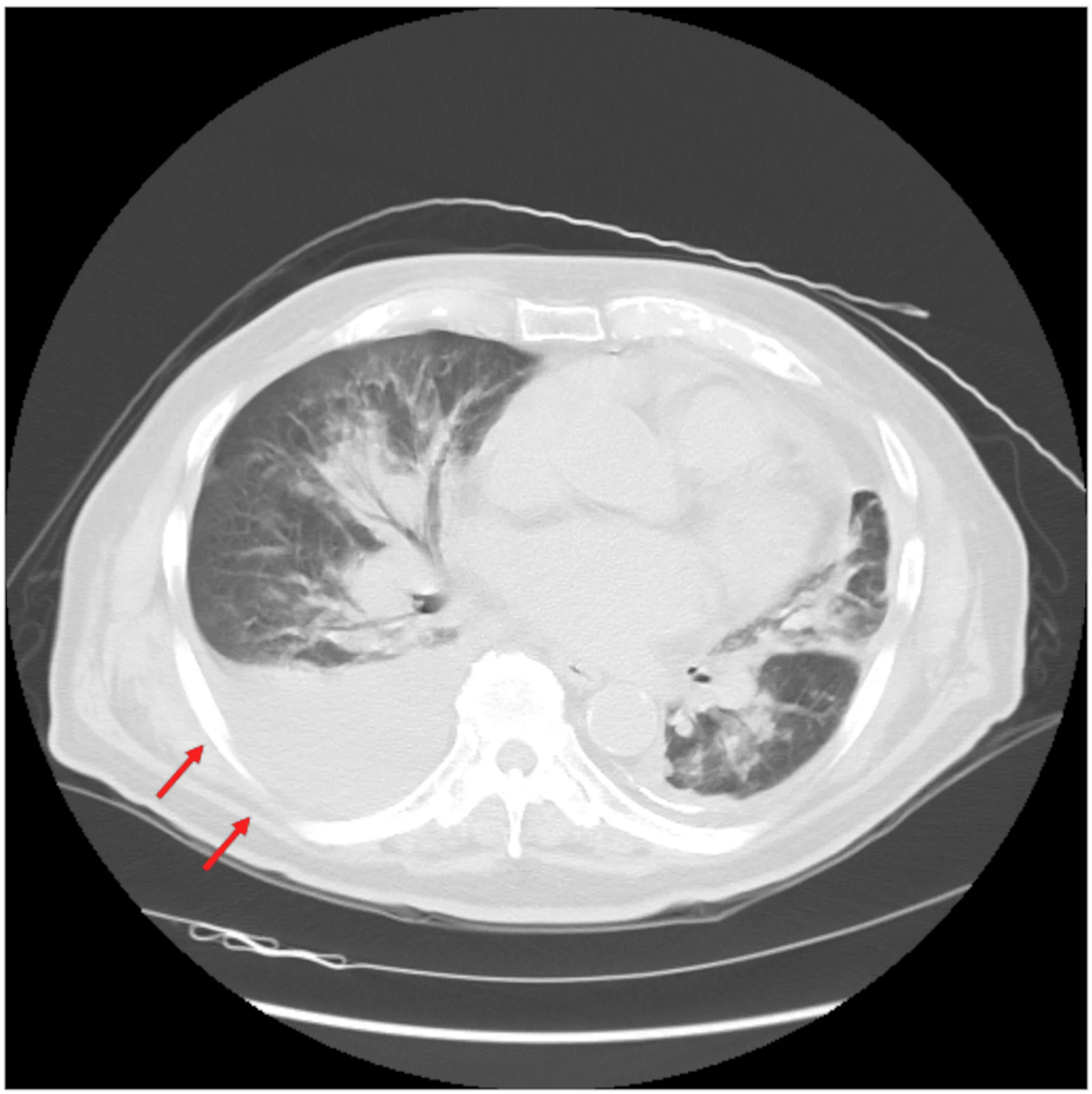
Chest computed tomography image Subsequent computed tomography revealed bilateral pleural effusions, greater on the right (arrows), accompanied by pulmonary congestion.

Electrocardiography showed atrial fibrillation (Figure 3).

**Figure 3:**
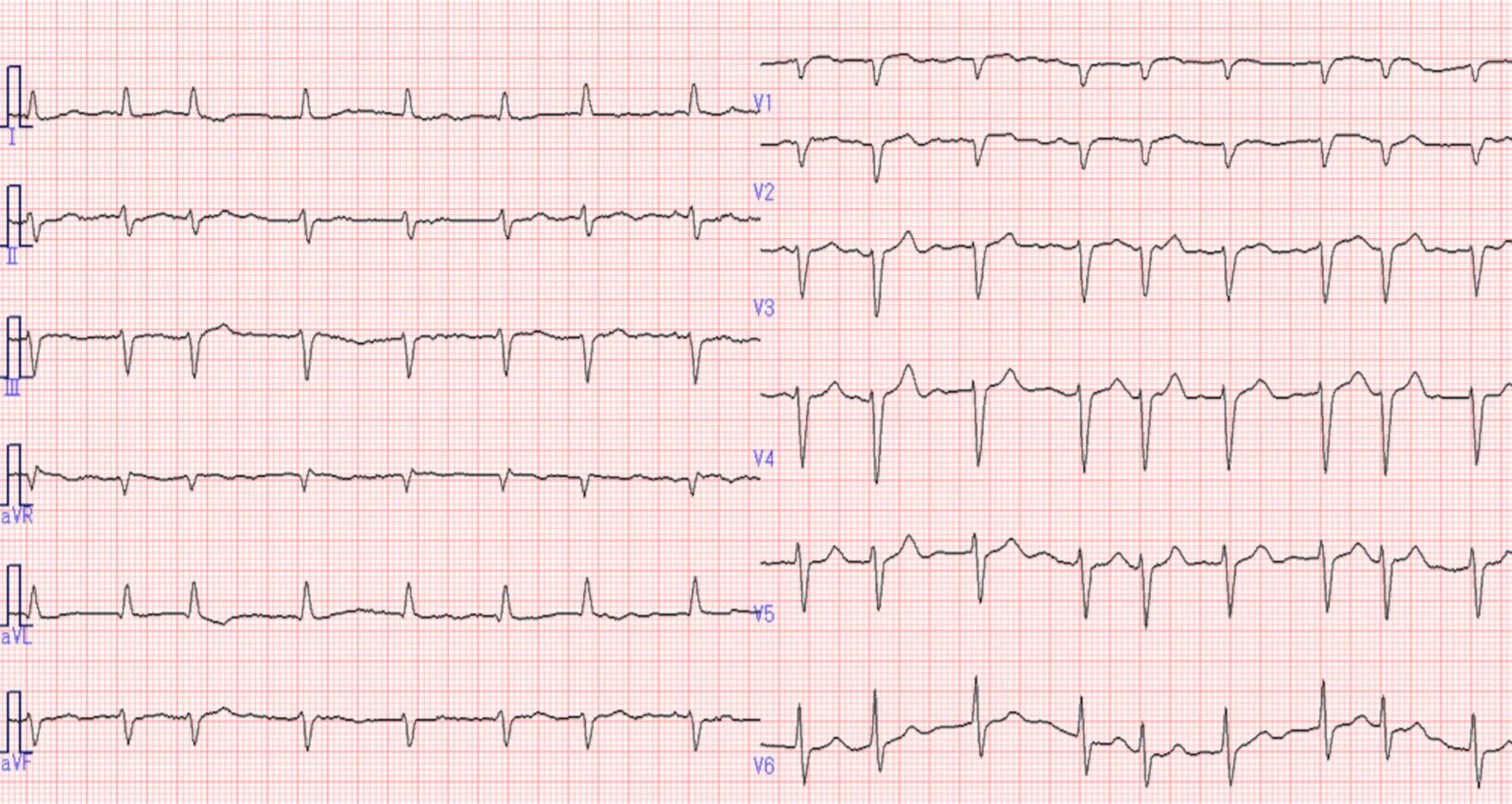
Electrocardiogram Electrocardiography showed atrial fibrillation.

Transthoracic echocardiography demonstrated a preserved ejection fraction (70%), mild left ventricular wall thickening (11 mm), an increased left atrial volume index (48.9 mL/m²), reduced septal and lateral e′ velocities (6.8 and 6.4 cm/s, respectively), an elevated E/e′ ratio of 16.7, an increased tricuspid regurgitation velocity (3.5-4.2 m/s), and mild mitral and tricuspid regurgitation, supporting elevated left ventricular filling pressure. Measurements were averaged over several cardiac cycles because of an irregular RR interval. The inferior vena cava was dilated, measuring 24 mm and decreasing to 12 mm with inspiration (Figure 4).

**Figure 4:**
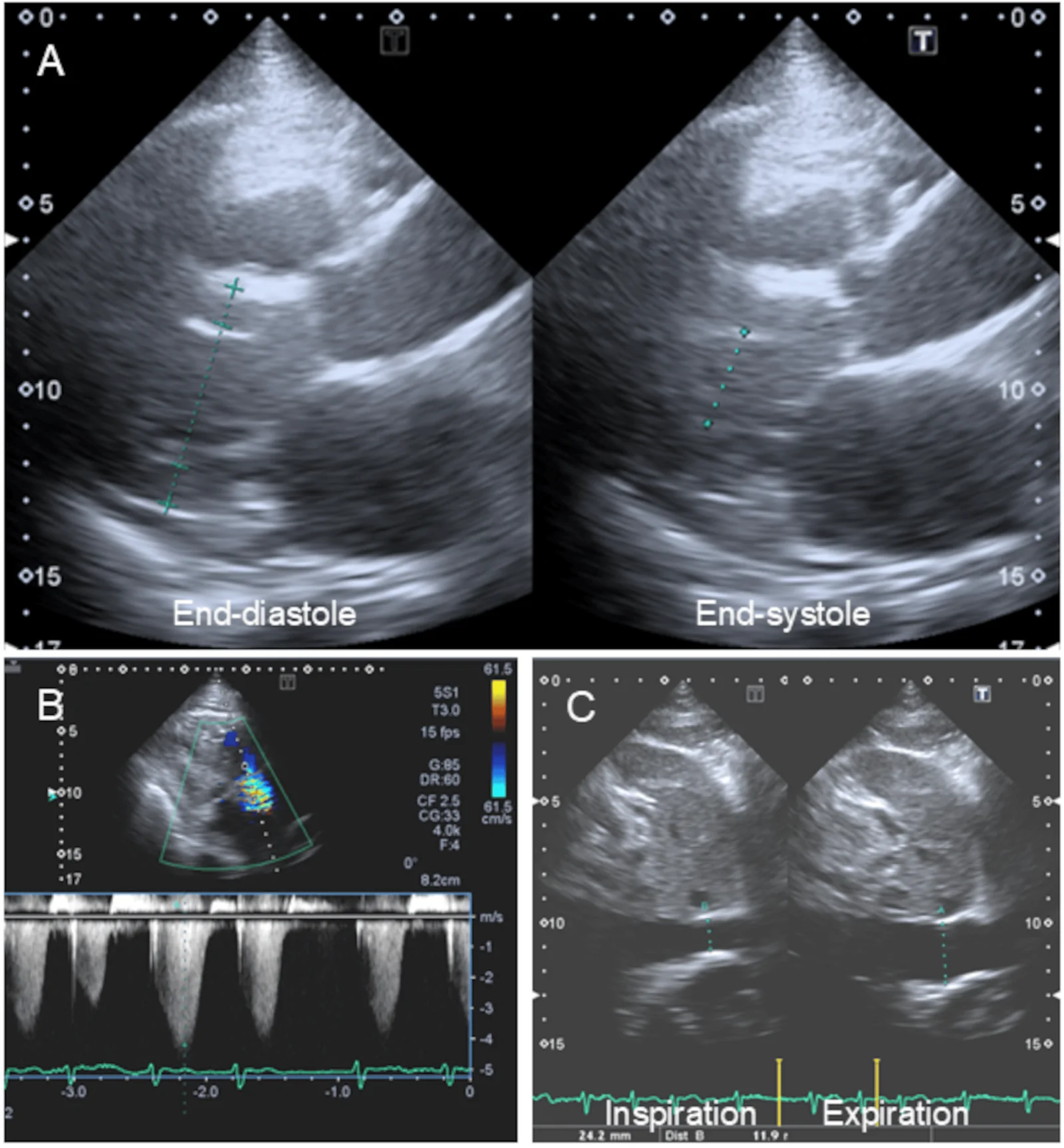
Transthoracic echocardiographic images Transthoracic echocardiography demonstrated preserved ejection fraction (70%), mild left ventricular wall thickening (11 mm) (A), and pulmonary hypertension (tricuspid regurgitation pressure gradient, 48–70 mmHg) (B). The inferior vena cava was dilated, measuring 24 mm and decreasing to 12 mm with inspiration (C).

He was diagnosed with decompensated HFpEF in the setting of atrial fibrillation. Given the hypokalemia with inappropriate renal potassium loss, Shakuyakukanzoto-induced pseudoaldosteronism was suspected as a potential contributor to the decompensation. Potential alternative contributors to decompensated HFpEF were also considered. There was no clear evidence of inadequate rate control of atrial fibrillation. Although troponin I was elevated, there were no reported ischemic symptoms or ECG changes suggestive of acute coronary syndrome. CRP was elevated, but there was no clear clinical evidence of infection. Shakuyakukanzoto was discontinued, and he received intravenous furosemide (20 mg) on the first day, followed by oral azosemide (30 mg/day), potassium supplementation, and spironolactone (50 mg/day). PRA and PAC measured on admission in the supine position, before initiation of diuretic therapy or spironolactone, were subsequently found to be 0.4 ng/mL/h and <4.0 pg/mL, respectively. These findings, together with hypokalemia and inappropriate renal potassium loss, supported the diagnosis of pseudoaldosteronism.

As shown in Figure 5, over approximately three weeks, serum potassium levels normalized, body weight decreased from 73 to 68 kg, and pleural effusion and pulmonary congestion resolved on chest radiography (Figure 1B), along with peripheral edema. NT-proBNP decreased to 1,889 pg/mL. Because blood pressure remained well controlled, sacubitril/valsartan, which had replaced azilsartan, was subsequently tapered and discontinued after hospital day 18, and amlodipine was also reduced to 2.5 mg/day at hospital day 24. Regarding serum potassium, supplementation was discontinued after hospital day 9, and spironolactone was tapered and subsequently discontinued after hospital day 22. Serum potassium remained stable at hospital day 28.

**Figure 5:**
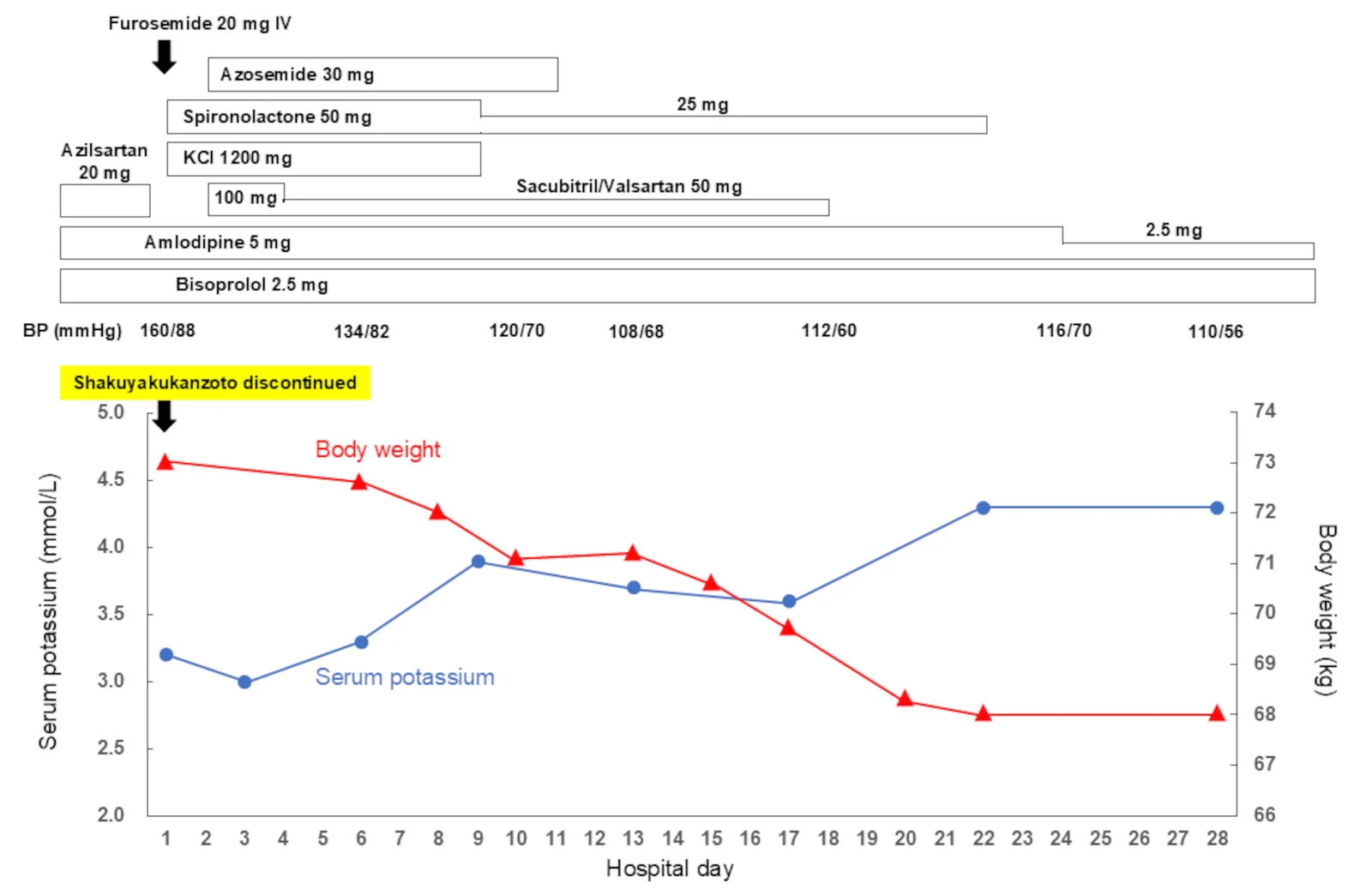
Clinical course Over approximately three weeks, serum potassium levels normalized, and body weight decreased from 73 to 68 kg. BP, blood pressure.

The patient continued cardiac rehabilitation and was discharged six weeks after admission. At the 10-week follow-up, he remained clinically stable without recurrence of heart failure.

## Discussion

In this report, we describe an elderly man in whom glycyrrhizin-induced pseudoaldosteronism contributed to decompensated HFpEF. Discontinuation of Shakuyakukanzoto, together with potassium supplementation, diuretic therapy, and spironolactone, resulted in resolution of hypokalemia and congestive findings and allowed reduction of antihypertensive medications.

At presentation, we suspected pseudoaldosteronism based on the combination of hypokalemia, inappropriate renal potassium loss, and long-term use of Shakuyakukanzoto. PRA and PAC measured on admission were 0.4 ng/mL/h and <4.0 pg/mL, respectively, while the patient was receiving azilsartan and bisoprolol. Although these medications may have influenced PRA and PAC levels, these findings and the clinical features supported the diagnosis of pseudoaldosteronism.

Glycyrrhizin and its active metabolites inhibit 11β-HSD2, allowing cortisol to activate mineralocorticoid receptors in the distal nephron, thereby promoting sodium retention and potassium excretion. Several studies have suggested that 11β-HSD2 activity declines with age [7,8], which may increase susceptibility to glycyrrhizin-induced mineralocorticoid effects. In the present case, this age-related decline may have contributed to the clinically apparent development of pseudoaldosteronism in this 94-year-old man after more than 10 years of Shakuyakukanzoto use, although the reason for its late manifestation remains uncertain because longitudinal clinical data before admission were unavailable.

The patient's hypokalemia and volume overload improved after withdrawal of Shakuyakukanzoto, together with potassium supplementation, diuretic therapy, and spironolactone. This temporal response was compatible with glycyrrhizin-associated pseudoaldosteronism contributing to decompensated HFpEF. However, the individual contribution of Shakuyakukanzoto withdrawal to the subsequent improvement cannot be determined because several treatments were initiated concurrently.

Only a few reports, predominantly from Japan, have described heart failure as a clinical manifestation of pseudoaldosteronism, including cases in patients with underlying hypertrophic cardiomyopathy [9] or a history of myocardial infarction [10]. In the present case, the patient had preserved systolic function but had several clinical features of HFpEF, including advanced age, atrial fibrillation, impaired diastolic function, and increased ventricular wall thickness [11]. In patients with these features, even modest increases in intravascular volume may disproportionately elevate cardiac filling pressures despite preserved systolic function [6], potentially leading to pulmonary and systemic congestion. In this patient, the decompensation of HFpEF may have resulted from sodium and water retention caused by pseudoaldosteronism in the setting of limited cardiac tolerance to volume loading. Another notable finding was hyponatremia (serum sodium, 127 mmol/L) despite volume overload and the sodium-retaining effects of pseudoaldosteronism. The mechanism of hyponatremia could not be precisely determined because serum and urine osmolality were not measured. Impaired renal free-water excretion in the setting of heart failure may have contributed [12,13].

This case highlights the importance of considering glycyrrhizin-induced pseudoaldosteronism as a potentially reversible cause for the clinical manifestation of HFpEF. According to a recent study using the Japanese Adverse Drug Event Report database [4], Shakuyakukanzoto was the most frequently implicated herbal medicine, accounting for 42.9% of 210 cases of pseudoaldosteronism, followed by Yokukansan (22.4%). Importantly, pseudoaldosteronism may develop even after prolonged use; in our patient, it became clinically apparent after more than 10 years of Shakuyakukanzoto use. In patients presenting with heart failure, particularly those with hypokalemia and hypertension, careful assessment of glycyrrhizin-containing medication use and measurement of PRA and PAC may help identify pseudoaldosteronism as a potentially reversible cause of heart failure.

There are several limitations. First, because potassium supplementation, diuretic therapy, and spironolactone were administered concurrently with Shakuyakukanzoto withdrawal, the subsequent improvement cannot be attributed solely to drug withdrawal. Thus, the clinical course supports, but does not establish, a causal relationship between glycyrrhizin exposure and decompensated HFpEF. Second, 24-hour urinary potassium excretion was not measured, and renal potassium loss was assessed only by spot urinary potassium concentration. Finally, the absence of recurrent decompensation during 10 weeks of follow-up is reassuring but does not establish long-term prevention.

## Conclusions

In conclusion, glycyrrhizin-induced pseudoaldosteronism may contribute to decompensated HFpEF through sodium and water retention in patients with limited cardiac tolerance to volume loading. Measurement of PRA and PAC can help identify pseudoaldosteronism as a potentially reversible cause of heart failure. Recognition and discontinuation of the offending glycyrrhizin-containing medication can lead to rapid improvement in congestion and prevent recurrent heart failure.
